# Supplementary material for: Splice-Junction-Based Mapping of Alternative Isoforms in the Human Proteome
Source: Cell Rep. Author manuscript; Available in PMC 2020 Jan 15. (PMC6961840; doi:10.1016/j.celrep.2019.11.026)
Supplement: 2 [file NIHMS1546469-supplement-2.zip › DF1/PXD010154/AdrenalGland/AdrenalGland_12_COL12A1_SGPGTPLTGNAATEEER.pdf]

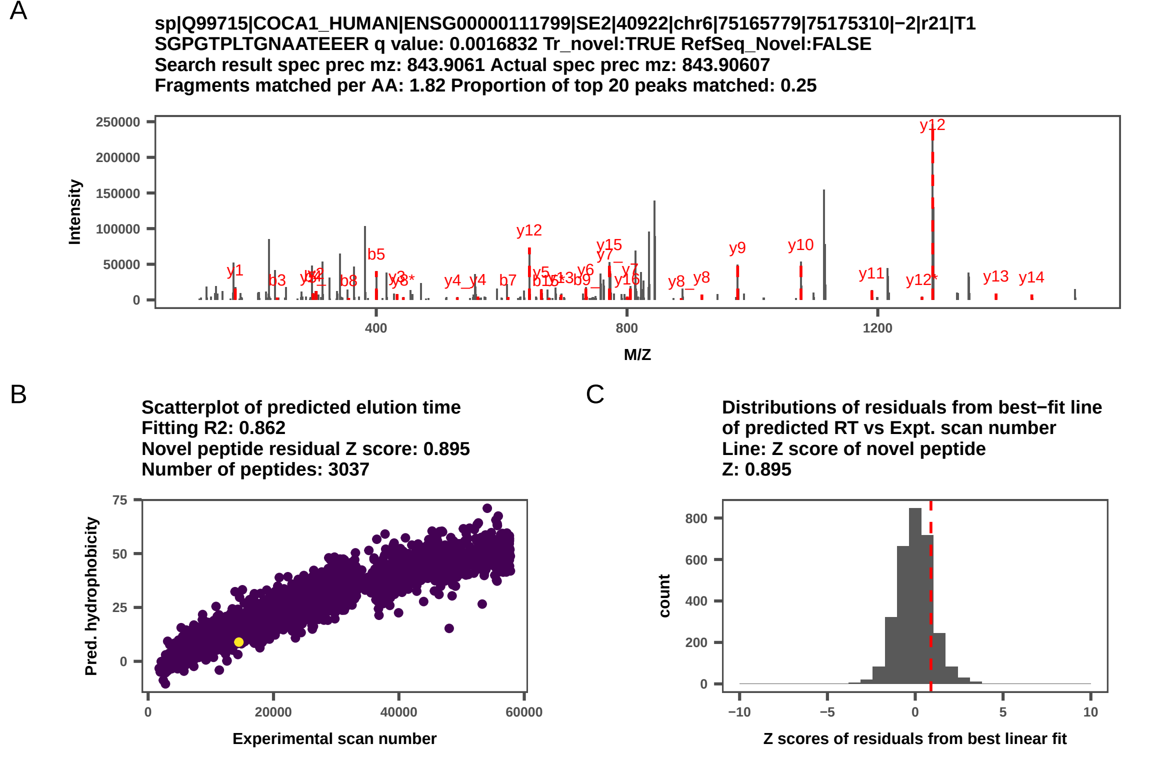
A
sp|Q99715|COCA1_HUMAN|ENSG00000111799|SE2|40922|chr6|75165779|75175310|−2|r21|T1
SGPGTPLTGNAATEEER q value: 0.0016832 Tr_novel:TRUE RefSeq_Novel:FALSE
Search result spec prec mz: 843.9061 Actual spec prec mz: 843.90607
Fragments matched per AA: 1.82 Proportion of top 20 peaks matched: 0.25
Intensity
250000
200000
150000
100000
50000
0
400
800
1200
M/Z
y1
b3
b5
b7
b8
y12
y15
y9
y10
y11
y12
y12*
y13
y14
B
Scatterplot of predicted elution time
Fitting R2: 0.862
Novel peptide residual Z score: 0.895
Number of peptides: 3037
Pred. hydrophobicity
75
50
25
0
0
20000
40000
60000
Experimental scan number
C
Distributions of residuals from best−fit line
of predicted RT vs Expt. scan number
Line: Z score of novel peptide
Z: 0.895
count
800
600
400
200
0
−10
−5
0
5
10
Z scores of residuals from best linear fit
